# Clinical Implications of the Age Threshold in the 2022 ACR/EULAR Classification Criteria for Takayasu Arteritis: A Real-World Study of Patients Aged over 61 Years

**DOI:** 10.3390/diagnostics16203414

**Published:** 2026-10-20

**Authors:** Jang Woo Ha, Oh Chan Kwon, Yong-Beom Park, Sang-Won Lee

**Affiliations:** 1 Division of Rheumatology, Department of Internal Medicine, Yongin Severance Hospital, Yonsei University College of Medicine, Yongin 16995, Republic of Korea; hjwnmk@yuhs.ac; 2 Division of Rheumatology, Department of Internal Medicine, Gangnam Severance Hospital, Yonsei University College of Medicine, Seoul 06273, Republic of Korea; ockwon@yuhs.ac; 3 Division of Rheumatology, Department of Internal Medicine, Yonsei University College of Medicine, Seoul 03722, Republic of Korea; 4 Institute for Immunology and Immunological Diseases, Yonsei University College of Medicine, Seoul 03722, Republic of Korea

**Keywords:** Takayasu arteritis, the 2022 criteria, age, unclassifiable, isolated aortitis

## Abstract

**Background/Objectives**: The age item of the 2022 American College of Rheumatology (ACR) and the European Alliance of Associations for Rheumatology (EULAR) criteria for Takayasu arteritis (TAK) (the 2022 ACR/EULAR criteria) limits the reclassification of TAK among patients aged over 61 who were classified as TAK according to the 1990 ACR criteria. Therefore, we evaluated the clinical significance of the age item of the 2022 ACR/EULAR criteria. **Methods**: We included 22 previously TAK-diagnosed patients aged over 61 (TAK patient aged >61 years) and 41 TAK-reclassified patients aged 50–60 (controls) in this study and retrospectively reviewed their medical records. We compared the satisfied items of the 2022 ACR/EULAR criteria between the two groups and further investigated whether these 22 TAK patients aged >61 years fulfilled the criteria for other relevant diseases. **Results**: In terms of absolute requirements, TAK patients aged >61 years were significantly older than TAK-reclassified patients aged 50–60. However, there were no further significant differences in the additional clinical and imaging criteria between the two groups. TAK patients aged >61 years exhibited a significantly higher frequency of ascending aorta involvement than TAK-reclassified patients aged 50–60 (18.2% vs. 0%, *p* = 0.012). Among the 22 TAK patients aged >61 years, ten were categorized as clinically isolated aortitis, and three of the ten patients with CIA could additionally be categorized as restrictively defined-isolated aortitis. **Conclusions**: Our findings support the need for external validation of the current age threshold in larger cohorts from different ethnic and geographical populations.

## 1. Introduction

Takayasu arteritis (TAK) is a large-vessel vasculitis that primarily affects the aorta and its major first branches and is characterized by granulomatous inflammation mediated by IL-6- and IL-17-mediated immune responses in its pathophysiology [1]. Because TAK often occurs among relatively younger patients compared to giant cell arteritis (GCA), another large vessel vasculitis, an age-related item has been included or mentioned in the classification criteria for TAK to date [2,3]. The classification criteria for TAK, which were suggested by the American College of Rheumatology (ACR) in 1990 (the 1990 ACR criteria or the previous criteria), include ‘age at disease onset ≤40 years’ as one of the six items [2]. The new classification criteria for TAK, which were proposed by a joint group of the ACR and the European Alliance of Associations for Rheumatology (EULAR) in 2022 (the 2022 ACR/EULAR criteria), include ‘age ≤60 years at diagnosis’ as an absolute requirement together with ‘evidence of vasculitis on imaging’ [3]. The 1990 ACR criteria can be applied to TAK-suspected patients aged over 61 years, and when three or more items of the criteria are met, TAK can be classified regardless of age. Conversely, when only two absolute requirements of the 2022 ACR/EULAR criteria are met, the age criterion is then applied. Therefore, based on the 2022 ACR/EULAR criteria, TAK cannot be classified among patients aged over 61 years, even though TAK is strongly suspected [2,3].

In real clinical settings, we may encounter two confusing situations. One is that patients previously diagnosed with TAK and classified as having TAK according to the 1990 ACR criteria can no longer be classified as TAK according to the 2022 ACR/EULAR criteria when they are aged over 61 years at diagnosis (TAK patients aged >61 years) [2,3,4]. The other is that although new patients meet the 2022 ACR/EULAR criteria, except for the age item, they can no longer be classified as TAK according to the 2022 ACR/EULAR criteria when they are aged over 61 years (TAK-suspected patients aged over 61) [4,5,6]. In this study, we evaluated the clinical significance of the age item of the 2022 ACR/EULAR criteria. We selected 22 TAK patients aged >61 years from the cohort of Korean patients with TAK and assessed the frequency of patients fulfilling each item of the 2022 ACR/EULAR criteria except for the age item. We compared the variables between 22 TAK patients aged >61 years and 41 TAK-reclassified patients aged 50–60 (controls). Additionally, we investigated whether these 22 TAK patients aged >61 years fulfilled the criteria for other relevant diseases and explored the clinical implications of these alternative classification categories.

## 2. Materials and Methods

### 2.1. Patient and Control Selection

We screened 145 patients enrolled in the cohort of Korean patients with TAK. We selected both TAK patients aged >61 years and TAK-reclassified patients aged 50–60 (controls) from the cohort of Korean patients with TAK and retrospectively reviewed their medical records. The inclusion criteria were as follows: (i) classification of TAK according to the 1990 ACR criteria for TAK [2], (ii) fulfilment of the 2022 ACR/EULAR criteria for TAK (except for the age item for the 22 TAK aged >61 years patients) [3], (iii) first diagnosis at this tertiary university hospital by vasculitis-specific rheumatologists from 2005 to 2023, (iv) presence of sufficient evidence of vasculitis in the aorta and its major first branches on imaging with related clinical manifestations at diagnosis [1,3], (v) absence of supporting evidence suggesting any other vasculitides such as GCA or medical conditions other than TAK at diagnosis [3,7], (vi) presence of sufficient medical records regarding clinical, laboratory, and radiological data at diagnosis, (vii) no coexisting serious medical conditions mimicking TAK such as malignancies or hospitalisation-requiring infectious diseases, and (viii) no previous exposure to immunosuppressive drugs or glucocorticoids for TAK treatment within 4 weeks before diagnosis. Finally, 22 TAK patients aged >61 years and 41 TAK-reclassified patients aged 50–60 (controls) were included in this study.

### 2.2. Ethics Disclosure

This study was approved by the Institutional Review Board (IRB) of Severance Hospital (Seoul, Republic of Korea, IRB No. 4-2022-1075) and was conducted in accordance with the Declaration of Helsinki. Given the retrospective design of the study and the use of anonymised patient data, the requirement for written informed consent was waived by the IRB.

### 2.3. Definitions of Patients

In this study, “diagnosis” refers to the clinical diagnosis established by vasculitis-specific rheumatologists, whereas “classification” refers to fulfilment of established classification criteria. (1) ‘TAK patients aged >61 years’ were defined as those aged over 61 years at diagnosis who were previously diagnosed with TAK, classified as having TAK according to the 1990 ACR criteria, and met the 2022 ACR/EULAR criteria except for the age item, which meant that those could not be reclassified as TAK according to the 2022 ACR/EULAR criteria due to the age item [2,3]. (2) ‘TAK-reclassified patients aged 50–60’ were defined as those aged 50–60 years at diagnosis who were previously diagnosed with TAK, classified as having TAK according to the 1990 ACR criteria, and could be reclassified as TAK according to the 2022 ACR/EULAR criteria [2,3]. These patients were selected as controls for the reinvestigation of the validity of the age cut-off of 61 years. (3) ‘TAK-suspected patients aged over 61′ were defined as those aged over 61 who were strongly suspected of TAK, however, who could not be classified as TAK according to the 2022 ACR/EULAR criteria due to the age item [3]. Throughout this study, age refers to age at diagnosis.

### 2.4. Vascular Sites Affected by TAK

Imaging methods approved in this study included the four modalities, such as conventional angiography, computed tomography (CT) angiography, F-18 fluorodeoxyglucose-positron emission tomography (FDG-PET), and magnetic resonance (MR) angiography at diagnosis [4,8]. We investigated the frequency of vascular sites affected by TAK according to the following items: (i) ascending aorta; (ii) aortic arch; (iii) descending aorta; (iv) right subclavian artery; (v) right common carotid artery; (vi) left common carotid artery; (vii) left subclavian artery; (viii) celiac axis; (ix) superior mesenteric artery; (x) inferior mesenteric artery; (xi) bilateral renal artery; and (xii) infra-renal aorta [8].

### 2.5. Suggested Diseases for Patients Aged over 61

In this study, given the clinical, laboratory, and radiological data at diagnosis, particularly the affected vascular sites including the aorta and its first major branches, we considered the following suggested diseases for both TAK patients aged >61 years and TAK-suspected patients aged over 61: GCA [1,7,8]; polyarteritis nodosa [1,9]; antineutrophil cytoplasmic antibody-associated vasculitis [1,10,11,12,13,14]; immune complex small vessel vasculitis [1,15,16]; Behcet’s syndrome [1,17,18]; Cogan’s syndrome [1,19]; clinically isolated aortitis (CIA)/restrictively defined-isolated aortitis (RD-IA) [1,20,21,22]; lupus vasculitis [1,23]; rheumatoid vasculitis [1,24]; sarcoid vasculitis [1,25]; hepatitis B virus (HBV)/hepatitis C virus (HCV)-associated vasculitis [1,26]; syphilis-associated vasculitis (aortitis) [1,27]; cancer-associated vasculitis [1,28]; IgG4-related disease [1,29]; tuberculosis-associated vasculitis [1,30]; and other infectious aortitis [1,31].

### 2.6. Definition of CIA and RD-IA

To avoid or minimise the confusion in the differential diagnoses, in this study, we proposed two types of idiopathic aortitis using the following operational definitions: CIA was defined as evidence of inflammation in the aorta regardless of the first branch involvement, whereas RD-IA was defined as only evidence of inflammation in the ascending aorta, aortic arch, and descending aorta without inflammation in the first aortic major branch, unlike typical CIA [20,21,22].

### 2.7. Statistical Analysis

All statistical analyses were performed using SPSS version 26 (IBM Corporation, Armonk, NY, USA) for Windows (Microsoft Corporation, Redmond, WA, USA). Continuous and categorical variables were expressed as medians (interquartile ranges [IQR]) and numbers (percentages). Significant differences between two categorical variables were analysed using the Chi-square and Fisher’s exact tests. The Mann–Whitney U test was used to compare significant differences between two continuous variables. A *p*-value <0.05 was considered to be statistically significant.

## 3. Results

### 3.1. Comparative Analysis Between TAK Patients Aged >61 Years and TAK-Reclassified Patients Aged 50–60

In terms of absolute requirements, TAK patients aged >61 years were significantly older than TAK-reclassified patients aged 50–60 (64.5 years vs. 55.0 years, *p* <0.001). In terms of additional clinical criteria, no significant differences were observed between the two groups. In terms of additional imaging criteria, all patients exhibited evidence of vasculitis in the aorta and its first major branches on imaging; however, there were no significant differences between the two groups (Table 1).

### 3.2. Comparative Analysis of Affected Vascular Sites Between TAK Patients Aged >61 Years and TAK-Reclassified Patients Aged 50–60

TAK patients aged >61 years exhibited a significantly higher frequency of ascending aorta involvement than TAK-reclassified patients aged 50–60 (18.2% vs. 0%, *p* = 0.012). However, among the remaining vascular sites, no significant differences in the distribution of radiological features of TAK involvement were observed between the two groups (Table 2).

### 3.3. Suggested Diseases for Patients Aged over 61

**Figure 1:**
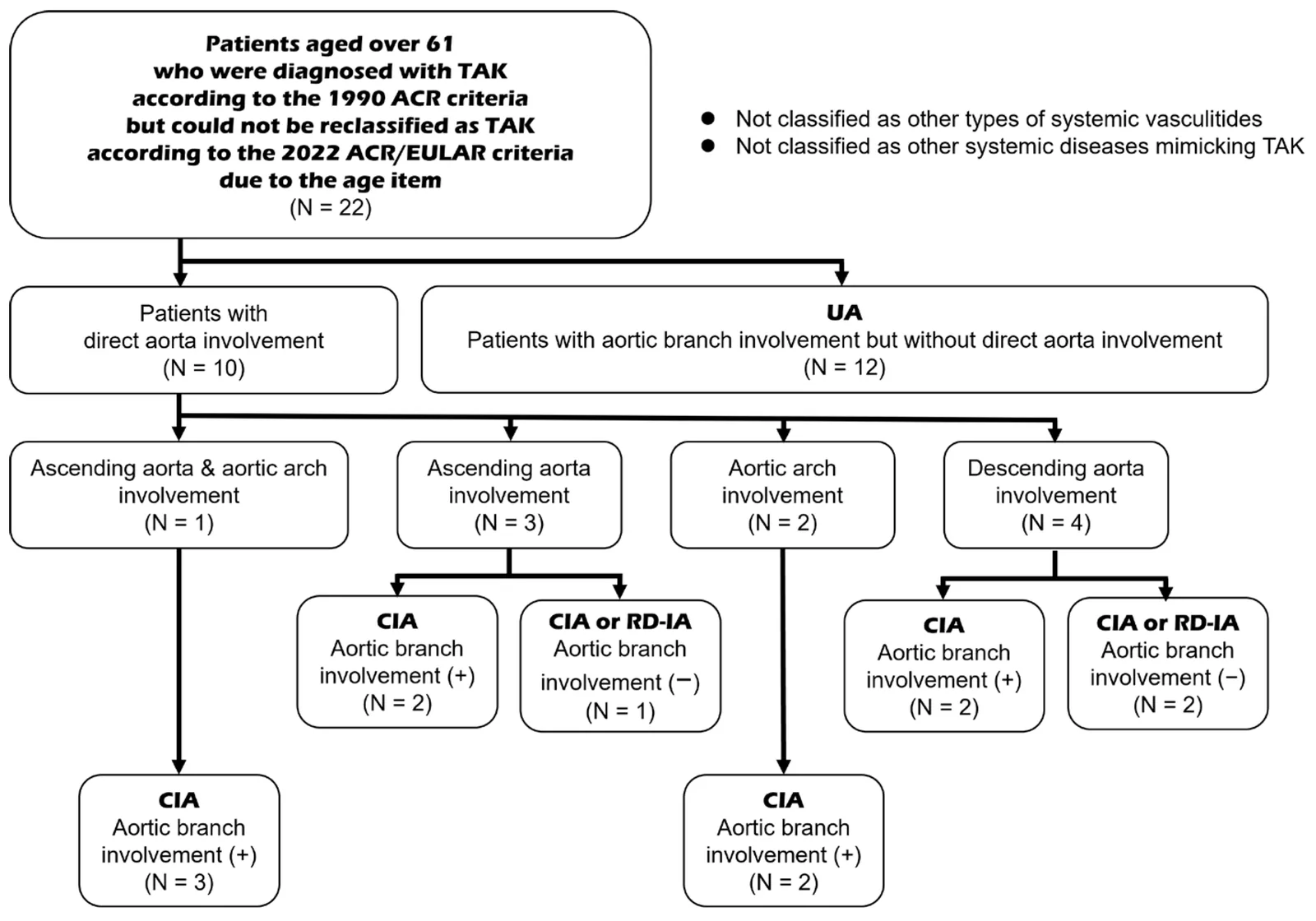
Algorithm for the classification of TAK, CIA, RD-IA, and UV. (+) indicates the presence of aortic branch involvement, whereas (−) indicates its absence. TAK: Takayasu arteritis; ACR: the American College of Rheumatology; EULAR: the European Alliance of Associations for Rheumatology; UV: unclassifiable vasculitis; CIA: clinically isolated aortitis; and RD-IA: restrictively defined-isolated aortitis.

Based on the clinical, laboratory, and radiological data recorded in the medical records and the sites of the involved vessels, we applied the diagnostic/classification criteria or the operational definition for each suggested disease to TAK patients aged >61 years. Among the 22 TAK patients aged >61 years, ten were categorized as CIA, and three of the ten patients were additionally categorized as RD-IA. However, none fulfilled the criteria for the remaining 15 suggested diseases (Table 3). Among the ten patients compatible with CIA/RD-IA, one, three, two, and four patients exhibited evidence of inflammation in the ascending aorta and aortic arch, ascending aorta, aortic arch, and descending aorta, respectively. Among the 10 patients, seven patients who exhibited evidence of inflammation in the aorta, as well as in the first aortic branches, were categorized as CIA, whereas the remaining three patients who had evidence of inflammation only in the aorta without inflammation in the first aortic branches were categorized as RD-IA (Figure 1). On the other hand, apart from ten patients compatible with CIA/RD-IA, 12 of the 22 did not fulfil any of the criteria for the other suggested diseases and were categorized as unclassifiable vasculitis (UV) because they had no evidence of inflammation in the aorta, although they had evidence of inflammation in the first aortic major branches [32] (Figure 1).

## 4. Discussion

The purpose of this study was to reconsider the medical situation in which patients aged over 61, who were diagnosed with TAK and classified as having TAK based on the 1990 ACR criteria, could no longer be reclassified as TAK due to the age item of the 2022 ACR/EULAR criteria [2,3]. Therefore, in this study, we defined a new concept of TAK patients for ‘TAK aged >61 years patients’ as follows: patients were defined as those aged over 61 years who were previously diagnosed with TAK and classified as having TAK according to the 1990 ACR criteria, and who met the 2022 ACR/EULAR criteria except for the age item, but who could not be reclassified as TAK according to the 2022 ACR/EULAR criteria due to the age item. We compared the variables between 22 TAK patients aged >61 years and 41 TAK-reclassified patients aged 50–60 (controls) and obtained two findings: One is that, except for the age item, there was no difference in the frequency of satisfied items of the 2022 ACR/EULAR criteria between TAK patients aged >61 years and TAK-reclassified patients aged 50–60. The other is that there were no significant differences in the vascular sites affected by TAK between the two groups, except for the ascending aortic involvement. However, given the small number of events and the comparison of multiple vascular territories, this isolated difference should not be interpreted as evidence of a specific vascular phenotype. With these results, we cautiously proposed that an absolute requirement of ‘age ≤60 years’ be changed to a more flexible item in actual clinical practice and suggest that the cut-off for the age-related absolute requirement of the 2022 ACR/EULAR criteria warrants external validation across different ethnic and geographical populations.

From the perspective of suggested diseases for TAK aged >61 years patients, this study revealed that only ten patients were categorized as CIA, and further three of them were categorized as RD-IA. In addition, the 12 patients who were not compatible with CIA/RD-IA were provisionally categorized as UV. Categorization of CIA and/or RD-IA may have potential clinical implications in terms of therapeutic approach and monitoring for progression to other systemic vasculitides such as GCA, granulomatosis with polyangiitis, or IgG4-RD [22]. Nonetheless, we would like to emphasise that patients who were not classifiable as TAK because they were aged over 61 years, despite fulfilling the remaining components of the 2022 ACR/EULAR criteria for TAK, should not necessarily be categorized as CIA/RD-IA, and their established clinical diagnosis and management should be maintained based on clinical judgment.

The medical situations in which the classification of TAK among patients aged over 61 years is difficult should be considered in two ways: ‘TAK patients aged >61 years’ and ‘TAK-suspected patients aged over 61’ [2,3]. First of all, categorization of these patients as UV for classification purposes may carry the following limitations: (i) a lack of theoretical reasons for the administered immunosuppressive drugs for the treatment of TAK [33]; (ii) a lack of evidence for active intervention in regular outpatient follow-up [34]; and (iii) a discontinuation of insurance benefits as the designation as a rare and incurable disease [35]. Therefore, for patients aged over 61 years at diagnosis who had been classified as TAK according to the 1990 ACR criteria, failure to fulfil the 2022 ACR/EULAR criteria solely because of age should not prompt a change in the clinical diagnosis of TAK.

Conversely, however, the initial application of the 2022 ACR/EULAR criteria to subjects who are suspected of TAK despite an age of 61 years or greater is not allowed in principle. Given the results of this study, a significant number of patients will likely be classified as UV, which will pose significant constraints in developing treatment strategies due to the limitations mentioned above [32]. Therefore, we assume that if the age cut-off for classifying TAK is adjusted upward to 70 years in patients aged over 61 years, a considerable number of patients aged over 61 years meeting the new criteria for TAK will be eligible for TAK classification [4], and we suggest that the cut-off for the age-related absolute requirement of the 2022 ACR/EULAR criteria needs external validation across different ethnic and geographical populations.

The reasons for selecting and including patients aged between 50 and 60 years who were classified as TAK according to the 2022 ACR/EULAR criteria as controls in this study were as follows: (i) of the 22 patients aged over 61 years, 21 were under 70 years of age. Therefore, patients aged 50 to 60 years, up to 10 years younger than the critical cut-off of 60 years, were selected as controls; (ii) TAK is characterized by a relatively high proportion of female patients. Therefore, given that the general age of menopause is around 50 years, to minimise the impact of menopause on clinical data at diagnosis, patients aged between 50 and 60 years were selected as controls [36]; and (iii) setting the age of the new criteria for TAK at 60 years or younger may be to differentiate it from GCA, but also from atherosclerosis, of which risk increases along with age. Given the age pattern of metabolic syndrome at 50–60 years [37], to minimise the impact of metabolic syndrome on atherosclerosis occurrence, patients aged between 50 and 60 years were included in this study as controls.

The strength of this study was that it was the first to demonstrate the clinical significance of the age item of the 2022 ACR/EULAR criteria for TAK among not only TAK patients aged >61 years but also TAK-suspected patients aged over 61 by comparing variables between TAK patients aged >61 years and TAK-reclassified patients aged 50–60.

This study has several limitations. The small number of patients, a single-centre study, and the retrospective study design were critical limitations of this study. In particular, the relatively small number of controls may have limited the statistical power to detect differences between the two groups. Furthermore, because fulfilment of the 2022 ACR/EULAR criteria except for the age item was an inclusion criterion for TAK aged >61 years patients, the observed similarity in the additional clinical and imaging criteria between the two groups may have been partly influenced by the study inclusion criteria. Therefore, these findings should be interpreted primarily as exploratory real-world observations. However, we are confident that this pilot study will have clinical significance in that it analytically proposed issues regarding the classification of both ‘TAK patients aged >61 years’ and ‘TAK-suspected patients aged over 61’. A future prospective multi-centric study with a large number of patients of various age groups of more or less than 60 years will provide more reliable information on a flexible age-related item for the classification of TAK.

## 5. Conclusions

In this study, we demonstrated no significant differences in the satisfied items of clinical and imaging criteria of the 2022 ACR/EULAR criteria, except for the age item, between TAK patients aged >61 years and TAK-reclassified patients aged 50–60 for the first time. Therefore, our findings support the need for external validation of the current age threshold in larger cohorts from different ethnic and geographical populations before considering its modification.

## Figures and Tables

**Table 1:** Comparative analysis of the satisfied items of the 2022 ACR/EULAR criteria between TAK patients aged >61 years and TAK-reclassified patients aged 50–60.

Age at Classification	TAK-Reclassified Patients Aged 50–60 (*n* = 41)	TAK Patients Aged >61 Years (*n* = 22)	*p*-Value
**Absolute requirements**			
Age ≤60 years	41 (100)	0 (0)	<0.001
Age (years)	55.0 (5.0)	64.5 (4.0)	<0.001
Evidence of vasculitis on imaging	41 (100)	22 (100)	1.000
**Additional clinical criteria**			
Female sex	37 (90.2)	19 (86.4)	0.687
Angina or ischemic cardiac pain	7 (17.1)	4 (18.2)	1.000
Arm or leg claudication	16 (39.0)	5 (22.7)	0.191
Vascular bruit	34 (82.9)	15 (68.2)	0.180
Reduced pulse in upper extremities	26 (63.4)	18 (81.8)	0.129
Carotid artery abnormality	19 (46.3)	11 (50.0)	0.782
Systolic blood pressure difference in arms ≥20 mmHg	41 (100)	21 (95.5)	0.349
**Additional imaging criteria**			
Number of affected arteries (aorta or its primary branches)			0.827
One artery	9 (22.0)	5 (22.7)	
Two arteries	14 (34.1)	9 (40.9)	
Three or more arteries	18 (43.9)	8 (36.4)	
Symmetric involvement of paired arteries	18 (43.9)	7 (31.8)	0.350
Abdominal aorta involvement with renal or mesenteric involvement	4 (9.8)	1 (4.5)	0.466
**Total scores**	10.0 (4.0)	9.5 (4.0)	0.327
**A total score of ≥5 points (N, (%))**	41 (100)	22 (100)	N/A

Values are expressed as number (percentage) or median (quartile). ACR: the American College of Rheumatology; EULAR: the European Alliance of Associations for Rheumatology; TAK: Takayasu arteritis; and N/A: not applicable.

**Table 2:** Comparative analysis of affected vascular sites between TAK patients aged >61 years and TAK-reclassified patients aged 50–60.

Age at Classification	TAK-Reclassified Patients Aged 50–60 (*n* = 41)	TAK Aged >61 Years Patients (*n* = 22)	*p*-Value
**Vascular sites affected (N, (%))**			
Ascending aorta	0 (0)	4 (18.2)	0.012
Aortic arch	13 (31.7)	5 (22.7)	0.564
Descending aorta	10 (24.4)	2 (9.1)	0.188
Right subclavian artery	10 (24.4)	7 (31.8)	0.562
Right common carotid artery	15 (36.6)	4 (18.2)	0.159
Left common carotid artery	20 (48.8)	9 (40.9)	0.550
Left subclavian artery	20 (48.8)	13 (59.1)	0.435
Celiac trunk	3 (7.3)	1 (4.5)	1.000
Superior mesenteric artery	3 (7.3)	2 (9.1)	1.000
Inferior mesenteric artery	0 (0)	0 (0)	N/A
Bilateral renal arteries	8 (19.5)	3 (13.6)	0.733

Values are expressed as number (percentage) or median (quartile). ACR: the American College of Rheumatology; EULAR: the European Alliance of Associations for Rheumatology; TAK: Takayasu arteritis; and N/A: not applicable.

**Table 3:** Suggested diseases for patients aged over 61 years who were no longer classified as TAK.

Suspected Diseases Other than TAK	References Corresponding to the Criteria or Evidence for Each Suspected Disease	Comment
Giant cell arteritis	[1,7,8]	None
Polyarteritis nodosa	[1,9]	None
ANCA-associated vasculitis	[1,10,11,12,13,14]	None
Immune complex small vessel vasculitis	[1,15,16]	None
Behcet’s syndrome	[1,17,18]	None
Cogan’s syndrome	[1,19]	None
CIA/RDIA	[1,20,21,22]	Ten/three patients
Lupus vasculitis	[1,23]	None
Rheumatoid vasculitis	[1,24]	None
Sarcoid vasculitis	[1,25]	None
HBV/HCV-associated vasculitis	[1,26]	None *
Syphilis-associated vasculitis (aortitis)	[1,27]	None
Cancer-associated vasculitis	[1,28]	None
IgG4-related disease	[1,29]	None
Tuberculosis-associated vasculitis	[1,30]	None **
Other infectious aortitis	[1,31]	None

* One patient had resolved HBV infection (HBsAg negative, anti-HBc positive, anti-HBs positive) but was proven to have no evidence of relapse at diagnosis. ** One patient had latent tuberculosis but was proven to have no evidence of its reactivation at diagnosis. TAK: Takayasu arteritis; ANCA: antineutrophil cytoplasmic antibody; RD-CIA: restrictively defined-clinically isolated aortitis; HBV: hepatitis B virus; HCV: hepatitis C virus; and IgG4: immunoglobulin G4.

## Data Availability

Data will be provided on request by the authors.

## Abbreviations

The following abbreviations are used in this manuscript:

ACR	the American College of Rheumatology
CIA	clinically isolated aortitis
CT	computed tomography
EULAR	the European Alliance of Associations for Rheumatology
FDG-PET	F-18 fluorodeoxyglucose-positron emission tomography
GCA	giant cell arteritis
HBV	hepatitis B virus
HCV	hepatitis C virus
IQR	interquartile ranges
IRB	the Institutional Review Board
MR	magnetic resonance
RD-IA	restrictively defined-isolated aortitis
TAK	Takayasu arteritis
UV	unclassifiable vasculitis
